# Energy of injection-induced seismicity predicted from *in-situ* experiments

**DOI:** 10.1038/s41598-019-41306-x

**Published:** 2019-03-21

**Authors:** Louis De Barros, Frédéric Cappa, Yves Guglielmi, Laure Duboeuf, Jean-Robert Grasso

**Affiliations:** 1 Université Côte d’Azur, CNRS, Observatoire de la Côte d’Azur, IRD, Géoazur, France; 2 Institut Universitaire de France, Paris, France; 3 Lawrence Berkeley National Laboratory, Berkeley, USA; 4 NORSAR, Gunnar Randers vei 15, PO Box 52, N2027 Kjeller, Norway; 5 Université Grenoble Alpes, CNRS, OSUG, IRD, Isterre, France

## Abstract

The ability to predict the magnitude of an earthquake caused by deep fluid injections is an important factor for assessing the safety of the reservoir storage and the seismic hazard. Here, we propose a new approach to evaluate the seismic energy released during fluid injection by integrating injection parameters, induced aseismic deformation, and the distance of earthquake sources from injection. We use data from ten injection experiments performed at a decameter scale into fault zones in limestone and shale formations. We observe that the seismic energy and the hydraulic energy similarly depend on the injected fluid volume (*V*), as they both scale as *V*^3/2^. They show, however, a large discrepancy, partly related to a large aseismic deformation. Therefore, to accurately predict the released seismic energy, aseismic deformation should be considered in the budget through the residual deformation measured at the injection. Alternatively, the minimal hypocentral distance from injection points and the critical fluid pressure for fault reactivation can be used for a better prediction of the seismic moment in the total compilation of earthquakes observed during these experiments. Complementary to the prediction based only on the injected fluid volume, our approach opens the possibility of using alternative monitoring parameters to improve traffic-light protocols for induced earthquakes and the regulation of operational injection activities.

## Introduction

Over the last decades, important increases in the seismicity rates have been observed in relation with anthropogenic activities, especially underground fluid injections and productions^1^. Recent examples include earthquakes related to wastewater disposal^1,2^, hydraulic fracturing^3^, CO_2_ sequestration^4^, gas storage and extraction^5^, and geothermal energy^6,7^. For instance, wastewater injections, associated with shale gas extraction, have induced earthquakes with magnitude up to 5.8 in Oklahoma (USA). In this area, the yearly rate of M_w_ ≥ 3 earthquakes has changed from 1.6/year over the past century to more than 850/year in 2015^2^. Geothermal activity may also induce large earthquakes, as the magnitude M_w_ = 3.4 earthquake felt in the Basel city, Switzerland^8^. A M_w_ = 5.4 earthquake is also suspected to be induced by geothermal activity, near Pohang (South Korea) in 2017, in a very low seismicity area^9,10^. Thus, a large number of studies indicate that anthropogenic activity increases the risk of earthquakes that can potentially damage surrounding infrastructures and impact population.

In order to understand and mitigate the seismic risk associated with fluid storage and extraction in deep reservoir formations, it is of crucial importance to anticipate the seismic energy induced by fluid perturbations^11^. Since the pioneering linear relationship proposed by McGarr^12,13^, it is now well accepted that the induced seismic moment (M_o_) scales with the injected fluid volume (*V*). However, several authors recently showed that the moment depends rather on *V*^3/2^ than on *V*. Indeed, Galis *et al*.^14^. Used earthquake dynamic rupture simulations to show that the rupture arrest along a fault pressurized by local fluid injection is controlled by a competition between injection pressure and tectonic prestress. Previously, by revisiting the seismogenic index^15^, Van der Elst *et al*.^16^ statistically found a *V*^3/2b^ correlation with the seismic moment, where *b* is the Gutenberg-Richter exponent. Similarly, Schultz *et al*.^17^ showed that the number of events induced in the Duverner play (Canada) scales with the volume, or identically^18^, that the seismic moment scales with *V*^3/2^.

Furthermore, recent observations, acquired either in the laboratory^19^, during *in-situ* injection experiments^20–22^ or at the reservoir scale^23^ showed that the seismic moment is overestimated by several orders of magnitude by the McGarr’s model^13^ prediction. One reason for this discrepancy is due to the assumption that every failure is seismic and confines within the pressurized volume. However, in many situations, only a small fraction of the deformation induced by fluid pressurization is observed to be seismogenic^6,7,20,21,24–27^. When considering together the seismic and aseismic motions, this discrepancy reduces significantly^28^. Thus, improving the anticipation of the seismic moment released during fluid injection requires an accurate quantification of the seismogenic context as well as the seismic and aseismic behavior of fault zones. The seismogenic index^15^ (see method for its definition), together with the injected volume or the pressure rate, has been used to this aim to forecast the evolution on the injection-induced earthquakes in Oklahoma^29,30^. This parameter empirically gathers information on the stress state, the fault network and its frictional properties. Therefore, despite its strong potential for forecast purpose, it does not bring insights on the physics of the induced seismicity process.

Moreover, previous works have also shown that the characteristics of induced seismicity depend not only on the injected volume, but also on the reservoir geological structure^17,31,32^, the depth of injection and the stress state^33–35^, the density and frictional properties of faults^26,36^ and the hydraulic properties of the reservoir/fault system^37,38^. Therefore, understanding induced seismicity only based on the measurement of the injected fluid volume can be problematic. To anticipate the released seismic energy induced by fluid perturbations at depth, quantifying the relationships between seismicity, injection parameters, rock deformation and reservoir structure is fundamental. Here, we focus on estimating what monitoring data contribute in the released seismicity, while considering the aseismic component of the deformation in the energy budget.

In this study, we have first acquired simultaneously measurements of fluid pressure, flow rate, deformation and seismicity during a series of ten *in-situ* injection experiments at a decameter scale in fault zones into permeable limestone^20^ and low-permeable shales^26^, accessed from underground laboratories at about 300 m depth. Such experiments have an intermediate scale between the laboratory and the reservoir. They benefit, therefore, from *in-situ* conditions and deals with the full complexity of the geological material responses. They also benefit from a proximal (m) and dense monitoring network, allowing a precise characterization of the geology, hydro-mechanical and seismic conditions. From those experiments, we then evaluated the relationships between the seismic moment, injection parameters and induced deformation for this unique quantitative data set. Through our investigations, we show that the seismic energy scales as the hydraulic energy with the injected fluid volume as *V*^3/2^ when the aseismic deformation is included in the energy budget. Furthermore, our model better predicts the seismic moment when the critical fluid pressure for fault reactivation and the minimal hypocentral distance from injection points are considered.

## Fluid Injection Experiments Into Fault Zones

**Figure 1:**
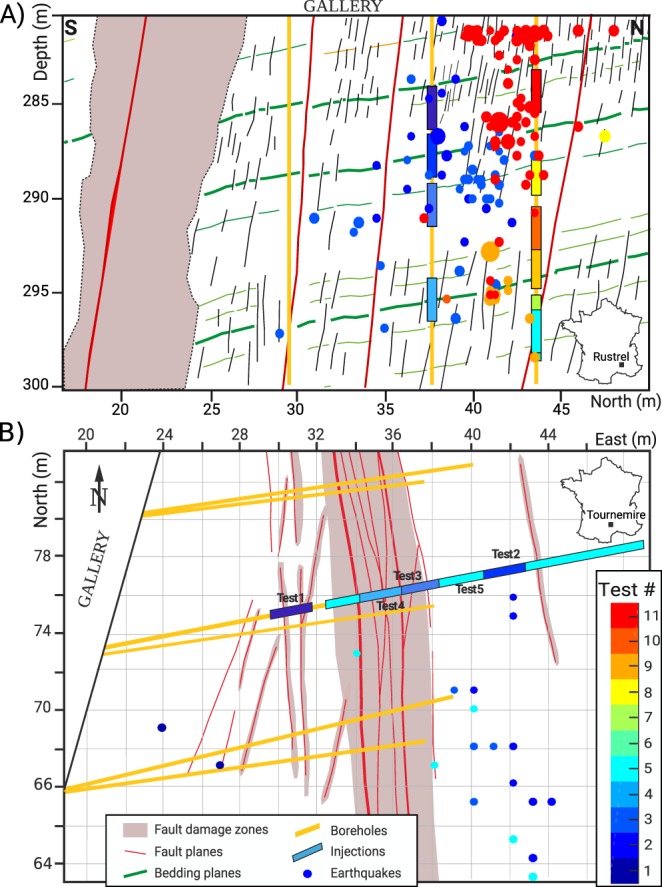
Experimental set-ups. Earthquake locations, colored by test number, and sized by magnitude projected in (**A**) a vertical cross-section view for the Rustrel (LSBB) experiment, and (**B**) a map view for the Tournemire experiment. The simplified geological structures (including faults identified from borehole logs, outcrops, and plugs), the monitoring boreholes and the injection locations are also indicated.

The aim of these controlled experiments of fluid injections was to reactivate selected existing geological discontinuities (fault segments, fractures, etc.) belonging to the extended damaged zone of kilometer-long faults. The response to two different geological contexts were explored. A first set of experiments^26^ took place in 2014 in shale rocks, accessed through the IRSN (Institut de Radioprotection et de Sureté Nucléaire) experimental platform of Tournemire (France). The second experiment^20^ was performed in 2015 into the highly permeable fractured limestone of the Low Noise Underground Laboratory of Rustrel (LSBB, France). Both underground laboratories are situated between 200 m and 300 m depth. At these depths, the stress state (2.1 MPa < σ_3_ < 3 MPa, and 4 MPa < σ_1_ < 6 MPa) favors shearing motions on the tested structures. From the tunnel walls, boreholes were drilled both for the injections and the monitoring network (see Fig. 1). Inside the injection boreholes, the SIMFIP (Step-rate Injection Method for Fracture *In-situ* Properties) probe^39^ was used to inject water into a sealed section of the borehole in order to induce mechanical failures on the geological structures that crosscut the injection chamber. Eleven and five different locations of the probe in Rustrel and Tournemire sites, respectively, allowed testing the responses of either secondary faults, minor fractures, part of the main fault core, or sub-horizontal bedding planes that separate layers with different facies. With a maximum injection pressure between 2.2 and 6.0 MPa, (i.e., values below σ_3_ plus the rock cohesion^24,40^), failures occurred only on optimally-oriented natural pre-existing structures, without inducing any new fractures.

In these experiments, a dense monitoring network surrounded these injections (14-to-22 accelerometers; see supplementary methods for more details on the experiment and the data analysis). In the injection chamber, the newly developed SIMFIP probe allowed the simultaneous measurements of the injected pressure, the flow-rate, and the 3D displacements of the tested structures^39^ (Supplementary Fig. S1). Results show that for the 5 Tournemire tests and 6 of the Rustrel tests, a residual displacement was observed at the end of the injections, which shows that failure was reached for those tests. We discard from this analysis the remaining 5 tests in Rustrel because of technical issues or lack of clear failure signatures, and one fully aseismic test from Tournemire. Ten tests are therefore used in the following, among which eight tests have a complete dataset. Around the injection, the seismicity was mainly recorded by a dense network of accelerometers^20,26^, which allow the detection, location and the magnitude computation of the seismic events. 34 and 215 events were recorded in the Tournemire and Rustrel experiments, respectively, with magnitude lying between −3.2 and −4.2. They are unevenly distributed among tests (see Fig. 1), as between none and 117 events were recorded. Their spatio-temporal distribution is also very heterogeneous. Particularly, no seismicity occurred close to the injection points despite mechanical failures monitored by the SIMFIP probe, highlighting a large aseismic component of the deformation^20,21,26^.

Therefore, these experiments lead to gather a unique monitoring dataset in a catalog (Supplementary Table S6) that includes injection parameters (pressure, flow-rate), 3D-displacement at the injection, and seismicity. Particularly, the aseismic deformation at the injection is here directly measured and compared to the seismic emissions. Finally, these data were recorded within two very different geological contexts, very low permeable shale and high permeable limestone.

## Results

### Energy budget

For each test, the maximum moment ${M}_{0}^{max}$ is the moment of the event with the largest magnitude, while the total moment ${M}_{0}^{tot}$ is the sum of the moments of all detected events. As the detection threshold is similar in both experiments, ${M}_{0}^{tot}$ corresponds to the cumulated moments of all events with magnitude M_w_ > −4^20,26^. According to Wyss^41^, ${M}_{0}^{tot}$ and ${M}_{0}^{max}$ are theoretically proportional. Both moments, together with the predicted models from van der Elst *et al*.^16^, Galis *et al*.^14^ and McGarr and Barbour^28^ can be converted to energy (see Methods). They are then compared with the hydraulic energy *Eh*, which represents the energy injected into the medium, and with the deformation moment ${M}_{0}^{def}$ (see Methods).

**Figure 2:**
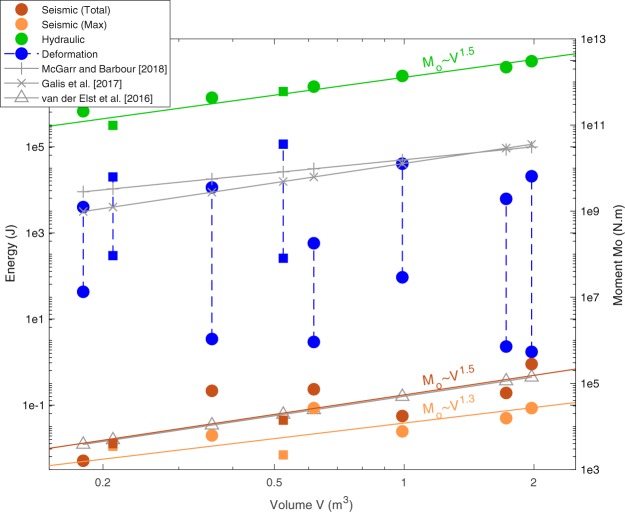
Seismic energy versus injected fluid volume. Maximum and total released seismic and hydraulic energy computed for the different tests performed in Rustrel (“o” symbols) and Tournemire (“□” symbols). Their best fitting trends are drawn as colored plain lines. Gray lines show the moment predictions converted to energy (upper bounds of ${M}_{0}^{tot}$ from Mc Garr and Barbour^28^, ${M}_{0}^{max}$ from Galis *et al*.^14^ and van der Elst *et al*.^16^).

We here observed that both the hydraulic moment *Eh* and the total moment ${M}_{0}^{tot}$ similarly scales with the volume to a power close to 3/2 (Fig. 2). It suggests that the seismic energy is a proportional fraction of the injected energy. A lower exponent is found in the relationship between ${M}_{0}^{max}$ and the volume. However, a 3/2 power is within the uncertainty range (σ = 0.26, inferred through bootstrap analysis). Therefore, these relationships agree with the theoretical predictions of Galis *et al*.^14^ and Van der Elst *et al*.^16^. However, there is a ~8 order of magnitude difference between the hydraulic and the observed seismic moments: the seismic energy only represents 4E-7% to 1.5E-5% of the energy brought during injections. This falls in the lower end of the reservoir responses, where the ratio seismic-to-hydraulic energy is observed to range between 8E-6% and 2%^42^.

A 5 order of magnitude difference is also noted between our observations and either Galis *et al*.^14^ or McGarr^13^ model. This discrepancy mainly comes from the fact that these models assume that all deformation is seismic, which is contradicted by observations^20,28^. The seismic moment is indeed only a small fraction (between 4e-3% and 1% in average) of the deformation moment, indicating that the induced deformation is mainly aseismic. Despite the very large uncertainties in the deformation moment, the predicting models of Galis *et al*.^14^ and McGarr^13^ models are at the upper bounds of these moments: they are therefore efficient to predict the total deformation, but not the released seismic moment.

The van der Elst^16^ model perfectly fits the ${M}_{0}^{tot}$ trend thanks to the use of the seismogenic index Σ as a free parameter. We find here a mean value of Σ = −3, consistently with reservoir measurements^42^ where Σ ranges between −10 and 1. This parameter characterizes the seismogenic state of a reservoir, and, hence, considers the amount of aseismic response. In details, this parameter varies between −2.7 and −3.6 in the Rustrel experiments, and between −3.2 and −4.9 in the Tournemire shale. Therefore, extrapolating this parameter from one injection to another may lead to errors in magnitude estimate greater than one, as it depends not only on the stress state, but also on the local fault architecture and on its frictional and hydromechanical properties. To summarize, the seismic energy and the injected energy show the same trend with the volume, but this latter parameter is not sufficient to accurately predict the released seismic energy, as the seismogenic state, including the aseismic motion, should be considered.

### Sensitivity of the seismicity to injection and reservoir parameters

**Figure 3:**
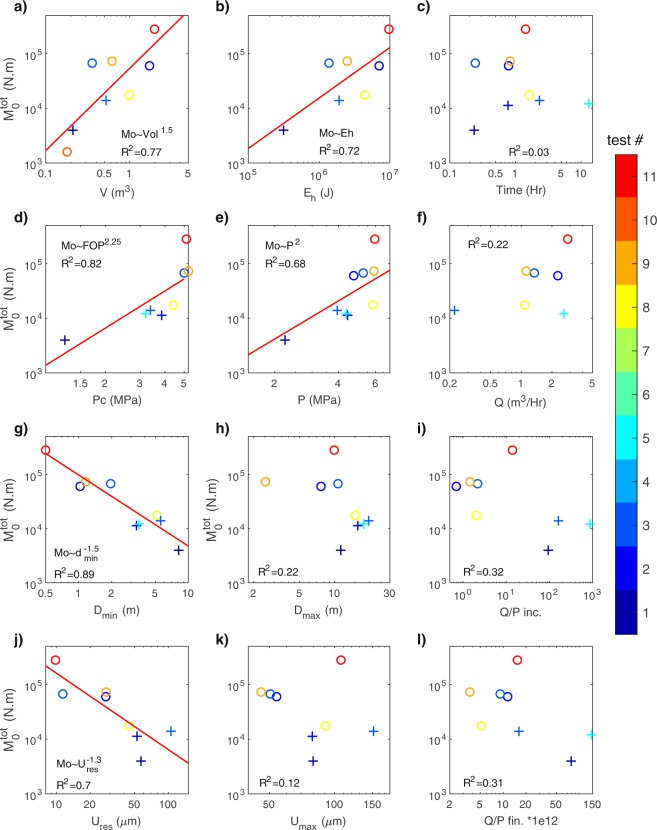
Released seismic moment and monitoring parameters. Total released seismic moment versus (**a**) Injected volume *V*. (**b**) Hydraulic energy *Eh*. (**c**) Injection duration time. (**d**) Critical Pressure *Pc*. (**e**) Maximum injection pressure *P*. (**f**) Maximum flowrate *Q*. (**g**) Minimal hypocentral distance from the injection *D*_*min*_. (**h**) Maximal hypocentral distance from the injection *D*_*max*_. (**i**) Ratio of the final over the initial Flowrate-Pressure ratio *Q*/*P*_*inc*_ (i.e. proxy for increase of permeability). (**j**) Residual displacement at the injection *U*_*res*_. (**k**) Maximum displacement at the injection *U*_*max*_. (**l**) Final Flowrate over Pressure ratio, i.e. proxy for final permeability *Q*/*P*_*fin*_. Colorscale refers to the test number in either Rustrel (“o” symbols) or Tournemire (“+” symbols) experiments. Red lines show the best fitting lines for which R² is greater than 0.6.

The seismic productivity, i.e. the released seismic energy, firstly scales with the volume. However, it may also depend on the reservoir properties, as fault responses to a fluid perturbation may be partly aseismic. We therefore explore the sensitivity of the released energy (or equivalently to the released moment) to the different parameters commonly used in reservoir engineering. Figure 3 shows the main results for the total seismic moment, with a fitting trend when the coefficient of determination *R*^2^ is above 0.6. The best fit solutions are obtained when using the total seismic moment although similar results were observed using either the maximum seismic moment (Supplementary Fig. S3) or the number of events (Supplementary Fig. S4).

#### Seismicity and injection parameters: injected volume (*V*), fluid pressure (*P*), flowrate (*Q*) and hydraulic energy (*Eh*)

As already stated, the total seismic moment is found to depend on the injected volume (Fig. 3a), with ${M}_{0}^{tot}\propto {V}^{3/2}\,({R}^{2}=0.77)$. The former also scales with the hydraulic energy (${M}_{0}^{tot}\propto Eh,\,{R}^{2}=0.72$, Fig. 3b). As expected, the more energy is injected, the larger the seismic energy is. In our experiments, but within two very different geological contexts, the seismic-to-injected energy ratio is constant at first order, and both scales with *V*^*3/2*^.

Seismic moment also scales with the maximum injection pressure *P* as ${M}_{0}^{tot}\propto {P}^{2}$ (Fig. 3e), but with a poorer fit (R^2^ = 0.68). This is not surprising, as the hydraulic energy also depends on pressure. However, the range of pressure variations is usually quite narrow, varying between the hydrostatic and the lithostatic pressures, which can reduce the effect of this parameter. As the stress state is not very different between both experiments (σ_3_ = 2.1 MPa and σ_3_ = 3 MPa), the correlation with a pressure-to-stress ratio (e.g. P/σ_3_), rather than with the pressure alone, cannot be tested.

Moreover, as the seismogenic index Σ^15^, seismic energy scales neither with the maximum flowrate nor with the injection duration (Fig. 3c,f). Similarly, no association between the flowrate and the seismicity is noticed for hydraulic fracturing cases^17^, contrary to wastewater disposal sites^37^. This discrepancy may come from the difference in injection rates^17^. Therefore, this parameter may be a control parameter when the fluid migration is dominated by poro-elastic diffusion, but not when large fault reactivation (or bulk fracturing) is induced.

#### Seismicity and total/residual displacements at the injec*tion* (*Umax/U*_*res*_)

The relationship between released seismic energy and displacement at the injection is very surprising (Fig. 3j,k). Seismic moment and maximum displacement at the injection point, which contains both poro-elastic and plastic motions, do not present a clear link. However, there is a clear, but counter-intuitive, relationship with the residual (i.e. plastic) displacement: ${M}_{0}^{tot}\propto {{U}_{res}}^{3/2}\,({R}^{2}=0.69)$. For a fast-rupture earthquake, a large deformation implies a large energy release^43^. However, as the displacement is here observed to be aseismic near the injection^20,26^, the released seismic energy is inversely related to the amount of aseismic deformation. Therefore, the larger the displacements are at the injection, the more energy is used in aseismic deformation, and less energy is available for seismic slips.

#### Seismicity and critical pressure (*Pc*)

The critical pressure is defined as the pressure required to reach the elasto-plastic failure limit. As failures are associated with an increase of permeability and of flowrate at constant pressure, it is the pressure for which the fractures start to hydraulically open above a critical stress^39,40^. Hence, it can be measured either as a change of trend in the pressure-flowrate response, by a change in the displacement direction at the injection, or by a loss of linearity in the displacement versus pressure relationship (Supplementary Methods and Fig. S2). Thus, this parameter quantifies the ability to reactivate a set of geological structures, depending on their orientation towards the stress state and on their frictional properties.

Here, we find a relationship (Fig. 3d) with the seismic moment: ${M}_{0}^{tot}\propto P{c}^{2.25}\,({R}^{2}=0.84)$. Therefore, the higher the pressure required to reactivate the structures is, the higher the seismic energy is. As for the pressure, *Pc* and the stress state or the stress deviator should be intrinsically linked, but the similarity in the regional stress field in our experiments does not allow exploring this correlation.

#### Seismicity and permeability

Assuming that the fluid flows in a set of fractures and that the permeability is linked to the mechanical opening of some of these fracture through the Darcy’s law, the hydraulic conductivity is proportional to the flowrate-over-pressure ratio (*Q/P*, see Methods). This ratio is measured at the beginning and at the end of every injection test, and its increase is related to the increase in hydraulic conductivity. While a 50-to-500 times permeability increase is observed for the Tournemire tests, Rustrel tests show no or much smaller permeability changes, with less than a 20-times increase. This is related to the very different hydraulic properties of carbonates and shales host rocks. Thus, despite a general trend showing a decrease of the released moment with the increasing permeability, there is no significant relationship between these quantities (Fig. 3i,l). Same results were observed in controlled injections^44^ in the Grimsel granite (Switzerland). Therefore, seismicity moment cannot be used to infer the permeability changes in reservoir monitoring. However, permeability and permeability changes may play a role in the aseismic deformation^45^ and in the spatio-temporal distribution of the seismicity^46^.

#### Seismicity and distance to the injection (*D*_*min*_)

An interesting observation in this series of experiments is the lack of seismicity close to the injections. All events were deported, with the closest events between 0.4 to 9 m from the injection well centers. Importantly, the seismic moment scales with the distance of the closest event to the injection (Fig. 3g), with ${M}_{0}^{tot}\propto {D}_{min}^{-1.5}$ and a very good fit (*R*^2^ = 0.89). Note that, within each test, the closest event is usually not the largest one nor the first one. Intuitively, the furthest the events are, the lowest the fluid and stress perturbations are and the smallest the released seismic energy is. As the deformation is seen to be aseismic at the injection, the distance *D*_*min*_ represents the size of the aseismic volume around the injection, which explains its scaling with the residual displacement (Supplementary Fig. S5). *D*_*min*_ also depends on the injected volume $({D}_{min}\propto {V}^{-1})$, and with a lower fit quality, on the injection pressure. The seismic moment also links with the mean hypocentral distance from the injection with a poorer fit, but not with the maximal distance to the injection (Fig. 3h).

### Multi-parameter relationships between induced moment and monitoring data

**Figure 4:**
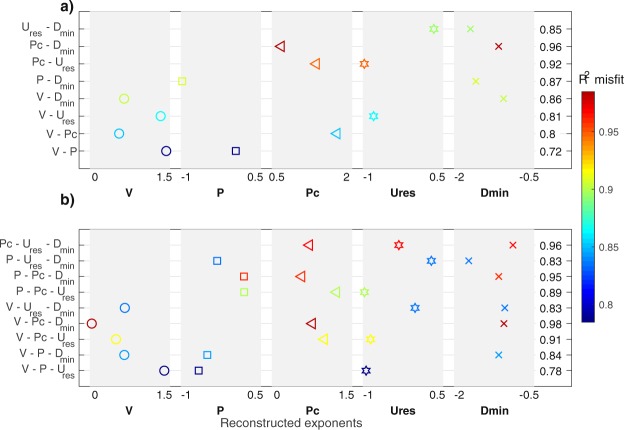
Multiparameters relationships for the released seismic moments. Exponents in the relationships (Eq. 1) between ${M}_{0}^{tot}$ and (**a**) 2 parameters or (**b**) 3 parameters, among the volume *V*, the pressure *P*, the critical pressure *Pc*, the residual displacement *U*_*res*_ or the minimal hypocentral distance *D*_*inj*_. The set of parameters that are jointly considered are given on the y-axis, left of the figures. The fit quality, expressed as R² coefficient is given by the color scale and by the number on the right of the figures.

Seismic moments correlate, not only with the injected volume, but also with the injection pressure, the residual displacement, the critical pressure, and the minimal hypocentral distance to the injection. On the contrary, the seismic moment depends neither on the injection duration, on the flowrate or on the permeability. So far, we looked for a direct correlation between the seismic moment and a single parameter, as if the monitoring parameters were independent from each other. However, trade-offs exist among them (Supplementary Fig. S5). That is why we now investigate the relationships of ${M}_{O}^{tot}$ with 2 to 3 parameters together. As all the relationships are power functions, this problem reduces to a linear inverse problem in a log-log scale, assuming a relationship of the form:

1 $$\begin{array}{c}{\rm{l}}{\rm{o}}{\rm{g}}({M}_{0}^{tot})=a+b\,{\rm{l}}{\rm{o}}{\rm{g}}(V)+c\,{\rm{l}}{\rm{o}}{\rm{g}}(P)+d\,{\rm{l}}{\rm{o}}{\rm{g}}\,{D}_{min}+e\,{\rm{l}}{\rm{o}}{\rm{g}}({U}_{res})+f\,{\rm{l}}{\rm{o}}{\rm{g}}(Pc)\,\end{array}$$

where *a* to *f* are the unknowns to determine. Some of these parameters are fixed to 0, in order to explore all the possible combinations of 2 or 3 parameters that allow the reconstruction of ${M}_{0}^{tot}$ (Fig. 4).

Among the relationships that include the volume, the following one leads to a good fit (R² = 0.8):

2 $$\begin{array}{c}{M}_{0}^{tot}=A{V}^{{\textstyle \tfrac{3}{2}}}\,{P}^{{\textstyle \tfrac{2}{3}}}{U}_{res}^{-1}\end{array}$$

with *A* = 10^−5^. This relation is interesting, as the exponent of the volume is 3/2 like the hydraulic energy. As the same volume exponent was found for single parameter correlation, it means that there is no trade-off between the volume and either the pressure or the residual deformation. The seismicity increases with the volume, but it is counterbalanced by the pressure and the deformation at the injection to take into account the non-seismic processes. Comparing this relationship with the seismogenic index^15^ leads to:

3 $$\begin{array}{c}\sum \,=\,C-\frac{4}{9b}\,\mathrm{log}(P)-\frac{2}{3b}\,\mathrm{log}({U}_{res})\end{array}$$

where *b* is the Gutenberg-Richter exponent and C is a constant with respect to the tested parameters. As Shapiro *et al*.^15^ found this index constant with respect to the injection parameters, a trade-off only depending on the seismotectonic context should exist between *P* and *U*_*res*_. However, this link cannot be clearly established here (Supplementary Fig. S5), even if a general trend exists among most of the tests. No other relations are found to have a *V*^3/2^ dependence, because of the trade-off between *V* and *D*_*min*_ (Supplementary Fig. S5). As the plastic displacement *U*_*res*_ is not an easily measurable parameter during an injection phase, such relationship might be difficult to use, except if a SIMFIP 3D strainmeter is set at the injection^39^.

The relationship leading to the best fit, with a minimal number of unknowns, does not depend on the injected volume, but only on *Pc* and *D*_*min*_:

4 $$\begin{array}{c}{M}_{0}^{tot}=A\,{D}_{min}^{-1}\,P{c}^{{\textstyle \tfrac{3}{4}}}\end{array}$$

with *A* very close to 1. This relationship, with only 2 parameters, leads to a near perfect fit (R² = 0.99). Therefore, the measurements of the critical pressure and of the minimal hypocentral distance from the injection allow a precise estimation of the induced seismic moments, at least in our experiments.

## Discussion

In this study, we observed that the released seismic moment is linearly related to the hydraulic energy, and both scale as *V*^3/2^, at first order. This result agrees with other recent results from Galis *et al*.^14^ and van der Elst *et al*.^16^, but not with the model brought by McGarr^13^. This discrepancy may come from the geometry of fluid pressure diffusion. Indeed, a 3D bulk diffusion will lead to a scaling with *V*, while a diffusion through a fault network, as observed in our experiments, will result in ${M}_{0}^{tot}\propto {V}^{3/2}$ ^47^. In any cases, the injected volume is the main parameter that allows a quantification of the energy brought during the injection. However, moment predictions based only on a correlation with the volume overestimate the released moments, as they neglect that a large part of the deformation is aseismic^21,28^. In order to improve the prediction of the released seismic energy, the seismotectonic response of a reservoir (i.e., if the geological structures will respond either seismically or not) should be considered. We therefore explored the sensitivity of the induced seismic moment to the monitoring parameters during industrial fluid manipulations in reservoirs. The seismic moment does not depend on flowrate and permeability, but it scales with the volume, the maximum pressure, the plastic displacement at the injection, the minimal hypocenter distance to the injection, and the critical pressure. Other factors, such as the stress state, the depth of the injection or the thermal difference between the injected fluid and the rock, were not measured in the experiments considered in this study, but may also change the seismic moments.

To predict released seismic moments, the hydraulic energy, which is directly correlated to the injected volume, should be counterbalanced by considering the contribution of the aseismic motion. The easiest way is to consider the residual displacement (*U*_*res*_), which is directly correlated to the aseismic motion, as it is the dominant deformation behavior compared with the much smaller seismic slips occurring far from the injection points^20,26^. The residual displacement depends on the seismotectonic properties of the reservoirs, as it is correlated with the orientation of the fracture network toward the stress field, and on their frictional and strength properties. Alternatively, the seismic moment can be predicted using the critical pressure (*Pc*) and the minimum hypocentral distance to the injection (*D*_*min*_). The critical pressure *Pc* is related to the pressure required to reactivate faults. Therefore, it may also inform on the stress state, the orientation of the faults towards it, on the frictional parameters of the faults and on their interactions. *D*_*min*_ depends on the volume and allows a quantification of the injected energy (Supplementary Fig. S5). Besides, it is also a direct measurement of the aseismic motion at the injection, as it is the minimal size of the aseismic deforming zone and it strongly depends on the residual deformation *U*_*res*_ at the injection. This distance might be related to an earthquake nucleation length, but it is observed to be much larger than the seismic source size. As the aseismic slip is observed to be strongly dilatant at the injection, the fault walls might lose contact, which prevents seismic slips to occur. In this case, *D*_*min*_ might be a characteristic length for which the aseismic slip becomes predominant over the dilatancy. Therefore, *D*_*min*_ might carry information on the shear stress versus pressure level at distance from the injection. This parameter might also be controlled by structural heterogeneities. Indeed, seismicity occurs on slip-weakening patches^26^ belonging to structures where the perturbed effective stress state allows failures in shear. Such conditions on structures, frictional properties and stress state might not exist close to the injections. In this case, seismicity occurs on distant structures once they are sufficiently impacted by either the stress transferred from aseismic motion or the fluid diffusion^20,21,24^.

Our analysis synthetizes results from^10^ independent injection tests, which are representative of the contribution of seismic and aseismic behaviors at an intermediate scale between laboratory experiments and reservoir observations. At this scale, our results suggest new approaches to explore the physical processes of injection-induced seismicity. Because the scale of our experiments is much smaller than the scale of industrial injection, the upscaling remains an open question. However, at the reservoir scales, the deformation is also observed to be dominantly aseismic, as monitored seismic events are not large enough to explain observed deformation^6,25,27^. Moreover, the seismogenic index computed here are in the range of the ones observed at the reservoir scales, it therefore suggests that moment-volume relationships are similar, and that seismogenic context and processes might be similar. The relationships inferred here should be anyway properly tested at the reservoir scales. To do so, aseismic deformation monitoring, by either direct measurement^39^ or through seismic velocity changes^22^, should be improved to better characterize and anticipate the spectrum of reservoir seismogenic behaviors. Then, both relationships ${M}_{0}^{tot}=A\,{V}^{{\textstyle \tfrac{3}{2}}}\,{P}^{-{\textstyle \tfrac{2}{3}}}\,{U}_{res}^{-1}$ and/or ${M}_{0}^{tot}=A{D}_{min}^{-1}\,P{c}^{{\textstyle \tfrac{3}{4}}}$ can be easily tested, and might then be implemented to feed traffic-light warning systems of reservoir induced seismicity^1,11,30^, in order to substitute the predictions based solely on the injected volume.

In our experiments, the injection times and volumes are small, with quite high injection pressure. Large-scale reactivation of faults are the dominant processes, while fluid diffusion is very limited in the intact rock or in the lower permeability fractures^6^. Similar high injection rates are performed in geothermal area and in reservoir exploitation through hydraulic fracturing, while wastewater disposals mainly induce poro-elastic diffusion. The inferred correlations between seismic moments and some parameters may vary according to the involved processes. For example, we do not observe a relationship between released moments and flowrate, while such relations were described for the Oklahoma seismicity^37^. Finally, we performed our experiments in rocks (limestone and shale) with very different frictional and hydromechanical properties. The seismic moments are well predicted by relationships that are not directly dependent on the rock properties. Therefore, once the upscaling to reservoir scale is verified, the relationships might be valid for any geological settings, as the seismotectonic context is indirectly considered through easily measurable parameters.

Our data set of injection-induced seismicity measured into fault zones within low- and high-permeability rocks allows for a better understanding of the relationship between seismic moment, injection parameters and induced deformation. The experiments clearly show that (1) only a small fraction of the injection energy and of the deformation is seismic, and (2) most of the deformation is aseismic at the injection and within the immediate volume that surrounds it.

When the contribution of the aseismic deformation is included in the energy budget, we find that the hydraulic and seismic energies are proportional to *V*^3/2^ rather the conventional model that scales with *V*. Furthermore, control parameters for the seismic moments are the residual displacement in addition to the injection volume, or alternatively the critical fluid pressure for fault reactivation and the hypocentral distance relative to injection. Thus, the present study suggests that direct measurements of aseismic displacements, and estimation of minimal hypocentral distances relative to injection and of the critical pressures, should be considered to improve the prediction of the released seismic moment used in engineer-based risk assessment, traffic light warning system and mitigation strategies of injection-induced seismicity.

## Methods: Computation of Moments and Energies

Moment predictions using the injected volume:

- McGarr^13,28^: ${M}_{0}^{tot}\le 2\,\mu V$, with *μ* the shear modulus. This relationship quantifies the upper bound of the cumulative seismic moment, as it also includes the aseismic component of the deformation.
- Gallis *et al*.:^14^ ${M}_{0}^{max}=\gamma {V}^{3/2}$, with $\gamma =\frac{0.4255}{\sqrt{d{\tau }_{0}}}{(\frac{(K{\mu }_{d}}{h})}^{3/2}$.
- *μ*_*d*_ is the dynamic friction coefficient, *dτ*_0_ is the background stress drop, *K* is the bulk modulus, and *h* is the characteristic length of the intersection of the fault with the reservoir. *h* is arbitrarily set to 20 m, as the estimated size of the pressurized zone. Note that h = 80 km should be used for a good fit of our moment data.
- The Seismogenic index Σ was originally defined by Shapiro *et al*.^15^ as $\sum =\,\mathrm{log}\,N+b{M}_{w}-\,\mathrm{log}\,V.$ *N* is the number of events with magnitude greater than *M*_*w*_ and *b* is the Gutemberg Richter exponent. Reformulating this equation leads to $\sum =b{M}_{w}^{max}-\,\mathrm{log}\,V$, with ${M}_{w}^{max}$ the magnitude of the largest event. van der Elst *et al*.^16^. Therefore used the relationship: $\mathrm{log}({M}_{0}^{max})=(\frac{3}{2b})({\rm{\Sigma }}+\,\mathrm{log}\,V)+9.2$.
- The moment of the event with the largest magnitude $({M}_{0}^{max})$ is proportional to the cumulated moment $({M}_{0}^{tot})$:

${M}_{0}^{tot}=\frac{B}{1-B}{M}_{0}^{max}$, where $B=2b/3$ ^41^. Therefore, sensitivity to the injection and reservoir parameters should be similar for ${M}_{0}^{max}$ and ${M}_{0}^{tot}$.

### Hydraulic energy

This energy is computed as: *Eh* = ∫ P*Qdt*, where *P* is the fluid pressure, *Q* is the injection flowrate, and *t* is the time.

### Moment versus energy

The seismic moment can be converted to energy by the relationship: ${M}_{0}=\frac{\mu }{{\rm{\Delta }}\sigma }E$ ^48^ . *μ* is the shear modulus, and Δ*σ* is the stress drop. The later was estimated to be about 100 kPa^20,26^. Therefore:

$${M}_{0}=3e5E,\,{\rm{or}}\,{\rm{equivalently}}:logE=\frac{3}{2}{M}_{W}+3.6,$$

with *M*_*W*_ the seismic magnitude. This is consistent with the empirical relationship:^43^ $logE=\frac{3}{2}{M}_{W}+4.8$, which was derived for large earthquakes with stress drop of 1–5 MPa.

The seismic moments are directly computed, while hydraulic energy is first computed. Therefore, for sake the consistency between these measures, all the seismic moments could have been converted to energy, or conversely, we here choose to convert the hydraulic energy to an equivalent hydraulic moment.

### Deformation moment ${{\boldsymbol{M}}}_{{\bf{0}}}^{{\boldsymbol{d}}{\boldsymbol{e}}{\boldsymbol{f}}}$

For a shear motion *u*_*s*_ or a tensile motion *u*_*n*_ induced on a crack, the deformation moment can be expressed as^48^:

$${M}_{0}^{def}=\mu \int {u}_{s}\,dS\,{\rm{or}}\,{M}_{0}^{def}=3K\int {u}_{n}\,dS,\,{\rm{respectively}}.$$

*μ* is the shear modulus and K is the bulk modulus.

We can then assume that (1) the Lamé’s parameters are equal (*λ* = *μ*), which leads to *K* = 5*μ*/3, and (2) that the displacements are maximal at the injection with a linear decrease with distance. Therefore, ${M}_{0}^{def}=\mu \,{U}_{s}\,S/2$ for shear motion and ${M}_{0}^{def}=5\,\mu \,{U}_{n}\,S/2$ for tensile displacement, with *U*_*s*_ and *U*_*n*_ the displacement measured at the injection, and *S* the rupture surface.

Separating the shear and tensile motions from the displacements measured at the injection required to project them on a fracture with known orientations and it is likely that several faults are playing during injections, with complex motions. Therefore, we prefer to keep a conservative approach, by computing the minimum and maximum values of the deformation moment.

The residual displacement contains only plastic, mainly shear, motion. The maximal displacement might be due to elastic and plastic behavior, with a large normal motion. Therefore, in order to find an uncertainty range for ${M}_{0}^{def}$:

$$\mu \,{U}_{res}\,S/2 < {M}_{0}^{def} < 5\,\mu \,{U}_{max}\,S/2$$

Computing the surface area on which failures occurs is not trivial. We therefore assume that the seismic location can give extremum values for the surface *S*. Indeed, we can consider that all the seismicity is either (1) occurring on frictional patches within the deforming area, or (2) triggered by stress transfer outside the deforming area. Under this hypothesis, and assuming a circular surface, the surface *S* of the deforming area can be bounded by $S=\pi \,{D}_{\min }^{2}$ and $S=\pi \,{D}_{max}^{2}$, with *D*_*min*_ and *D*_*max*_ the minimal and maximal hypocentral distance to the injection well, respectively. Therefore, the deformation moment falls into the range:

$$\mu \,{U}_{res}\,\pi \,{D}_{min}^{2}/2\le {M}_{0}^{def}\le 5\,\mu \,{U}_{max}\,\pi {D}_{max}^{2}/2$$

These inequalities give the uncertainty range in Fig. 1.

## Supplementary information

Supplementary materials

## Data Availability

The data used in this article are summarized in the Supplementary Table S6. Raw and unprocessed data are available upon request at debarros@geoazur.unice.fr.
