# Supplementary material for: Energy of injection-induced seismicity predicted from in-situ experiments
Source: Sci Rep. 2019 Mar 21;9:4999. doi: 10.1038/s41598-019-41306-x (PMC6428893; doi:10.1038/s41598-019-41306-x)
Supplement: Supplementary file 1 — Supplementary materials [file 41598_2019_41306_MOESM1_ESM.pdf]

Supplementary materials for the article:

Energy of injection-induced seismicity predicted from in-situ experiments

Louis De Barros[1,*], Frédéric Cappa[1,2], Yves Guglielmi[3], Laure Duboeuf[4], and Jean-Robert Grasso[5]

[1] Université Côte d'Azur, CNRS, Observatoire de la Côte d'Azur, IRD, Géoazur, France
[2] Institut Universitaire de France, Paris, France
[3] Lawrence Berkeley National Laboratory, Berkeley, USA
[4] NORSAR, Gunnar Randers vei 15, PO Box 52, N2027 Kjeller, Norway
[5] Université Grenoble Alpes, CNRS, OSUG, IRD, Isterre, France

[*] Corresponding author: Louis De Barros (debarros@geoazur.unice.fr; (+33) 4 83 61 86 22)

This file contains:

- Supplementary methods: experiments and monitoring data analysis
- Supplementary figures S1 to S5
- Supplementary tables S6

Supplementary methods: Experiments and monitoring data analysis

Earthquake locations and moments

In the Tournemire experiment (see De Barros et al.[1] , for more details), 14 3-C accelerometers (PCB356B28, with flat response between 2Hz and 4kHz) were cemented inside 5 boreholes at short distance (<20m) around the injections. Seismic data were continuously recorded at a 10 kHz sampling rate, synchronously with the hydro-mechanical data. Events were detected through a STA/LTA algorithm, complemented by a template matching approach during the injection periods. Event location was performed by minimizing the difference between the measured and computed P- and S-wave travel times and P-wave polarization on a spatial grid (0.5 m grid space in the three directions). The theoretical data were computed in a homogeneous velocity model. Based on calibration shots, the location error is estimated to be less than 1.5 m (i.e. larger than the grid spacing). All events are projected on the same map view in Fig. 1a.

In the Rustrel experiment (see Duboeuf et al.[2] , for more details), the injection area was surrounded by a network backbone composed of 8 3-C accelerometers (PCB356B28, 2Hz - 4kHz) anchored in 4 vertical boreholes and 14 1-C (BK4383) set on the gallery floor. All sensors are less than 20 meters from the injections and were continuously recorded at 10 KHz. Additionally, 9 geophones (10 Hz) and 4 acoustic sensors (1Hz-10kHz) completed the seismic monitoring network at low and high frequency. Events were detected by a STA/LTA approach. Absolute and relative locations were simultaneously performed, using together P- and S-wave travel times and differential times between similar events. This inverse problem was solved on a grid, with a 0.2 m resolution. The uncertainty on the absolute location was computed to be less than 2 meters, and about 5-to-10 times smaller for the relative locations.

For both experiments, the moment was computed by fitting a Brune model in acceleration on the spectrum[3] i.e. with a ω^2 slope at low frequencies and a flat plateau at high frequency. Attenuation coefficients of Q=30 and Q=70 were used in Tournemire and Rustrel, respectively. To remove the effects of the radiation patterns, measures of moments and frequency corners were performed for all sensors, on P- and S- waves separately, before being averaged. These assumptions on the radiation patterns may lead to errors between -0.5 and 0.2[4]. However, as the network is surrounding the seismicity in the 3 dimensions, the errors from the radiation patterns should be minimized. Tests were performed on the attenuation and velocity model and on the methods (data in acceleration or displacement) shows that the relative magnitude errors between experiments is lower than -0.2[1, 2] . Therefore, adding the errors coming from 1) the mislocation, 2) the radiation pattern, 3) the velocity and attenuation model and 4) the used methods may lead to large uncertainties. However, the consistency in the moment computation in both sites lead to consistent values among events and tests. We are therefore confident in the relative moment values between events and experiments, even if absolute value mays suffer from large uncertainity. We refer the readers to De Barros et al. [1] and Duboeuf et al.[2] for more details in the data analysis.

Hydromechanical data

The SIMFIP (Step-Rate Injection Method for Fractures In-situ Properties hydromechanical probe) was used to perform the injections on both sites[5]. This probe allows capturing the coupled hydro-mechanical response of 2.4 m long intervals of the borehole isolated by two inflatable straddling packers. In this injection chamber, located around selected fractures or faults, changes in fluid pressure, fractures mechanical displacements, and temperature were simultaneously monitored with a respective accuracy of 0.1 kPa, 3 µm and 0.1 °C. The flowrate is directly measured at the fluid injection

pump with a 0.1 L/s accuracy. The fault displacements are measured with a three-dimensional extensometer which is composed of two un-deformable rings anchored above and below the tested fractures in the borehole. These two rings are connected by an array of six deformable tubes instrumented with fiber optic Bragg strain gauges. Once the sensor is anchored, it is completely independent from the straddled system of the probe. Thus, a fracture movement eventually caused by the chamber pressurization will produce a relative displacement between the upper and the lower (considered fixed) anchors inducing deformations of the tubes. An algorithm allows to inverse the tubes strains into the relative three dimensional displacement of one fault compartment toward the other one[5]. An example of monitoring data (test 3, Rustrel), including pressure, flowrate, the three components of the deformation and the seismic occurrence is shown Fig. S1.

Displacements ($\mathbf{U_{max}}$, $\mathbf{U_{res}}$) and critical pressure Pc

The maximum displacement U_{max} is de fined as the maximum of the norm of the displacement, while the residual displacement U_{res} is the displacement norm after the injection end. While the first one may contain both plastic and elastic behavior, the latter is only related to plastic failures.
The critical pressure Pc is estimated by conducting a pressure step-rate test in a borehole. It is a test where the injection rate is increased step by step in order to stimulate a fracture or a fault, as at the beginning of the injection in Fig. S1. At each step, the flow rate is increased and held constant for the same amount of time. The critical pressure is signaled on a graph of flowrate-vs-pressure as the point where large increases in flow rate provide small increases in pressure (see Fig. S2). It can also be also measured in a 3D displacement graph, as it corresponds to the pressure for which the deformation switch from a (pseudo-)linear elastic behavior to a plastic one, indicated by a change in the amplitude and direction of the deformation (see Fig. S2). This parameter was also referred as FOP (Fault Opening Pressure) in Guglielmi et al. [5], where more details on its measure can be found.

Permeability estimation

The fluid is assumed to flow in a set of fractures. The permeability k is related to the hydraulic aperture bh by the cubic law[6]:

$$k = \frac{bh^3}{6s},$$

with s the fracture spring. The hydraulic aperture is given by:

$$bh = -\left(\frac{12\,\mu_f \Delta Q}{w\,\Delta P}\right)^{1/3}$$

with μ_f the water viscosity, w the fault width, ΔP the pressure change and ΔQ the flowrate change. Combining these two equations leads to $k \propto \frac{\Delta Q}{\Delta P}$.

Bibliography

[1] De Barros, L. et al., Fault structure, stress, or pressure control of the seismicity in shale? Insights from a controlled experiment of fluid-induced fault reactivation. ***Journal of geophysical Research: Solid Earth*** **121**(6), 4506-4522, 2016.

[2] Duboeuf, L., De Barros, L., Cappa, F., Guglielmi, Y., Deschamps, A., & Seguy S. Aseismic motions drive a sparse seismicity during fluid injections into a fractured zone in a **carbonate reservoir.** ***Journal of Geophysical Research: Solid Earth*** **122**(10), 8285-8304 (2017).

[3] Boore, D. M. Stochastic simulation of high-frequency ground motions based on seismological models of the radiated spectra. ***Bull. Seismol. Soc. Am.*** **73**(6), 1865–1894 (1983).

[4] Daniel, G. Bias in magnitude for earthquakes with unknown focal mechanism, ***Geophysical Prospecting*** **62**, 848–861, (2014).

[5] Guglielmi, Y., et al., ISRM suggested method for step-rate injection method for fracture in-situ properties (SIMFIP): Using a 3-components borehole deformation sensor. In Ulusay R. (eds) **The ISRM Suggested methods for rock charcaterization, Testing and Monitoring: 2007-2014**, 179-186, Springer (2013).

[6] Witherspoon, P., Wang, J., Iwai, K., & Gale, J., Validity of cubic law for fluid flow in a deformable rock fracture, ***Water resources research*** **16**(6), 1016-1024, (1980).

Figure S1: Example of monitoring data recorded during an injection

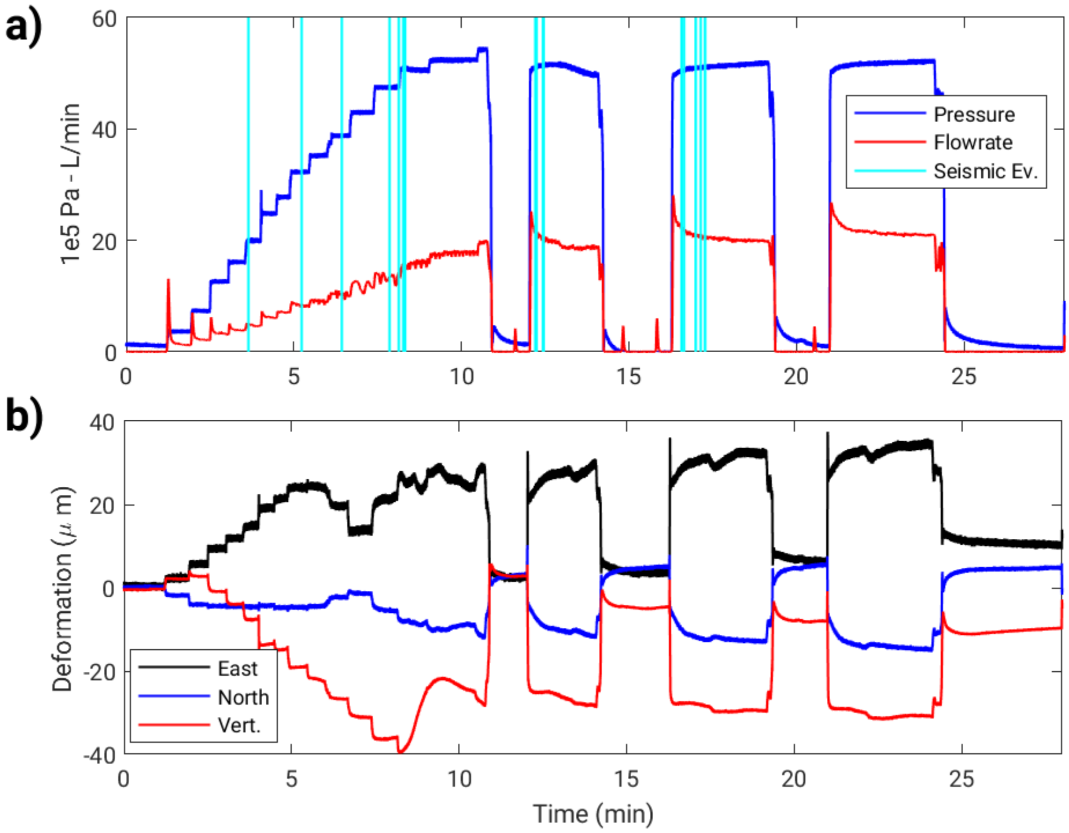

Figure S1: *Example of raw monitoring data recorded during an injection test (Test 2, Rustrel). a) Pressure, Flowrate and seismic occurrence. b) Three components (East, North and Vertical) of the deformation recorded at the injection point. Note that the displacement does not come back to 0 after the injection end, indicating a residual displacement.*

Figure S2: Measure of the critical pressure

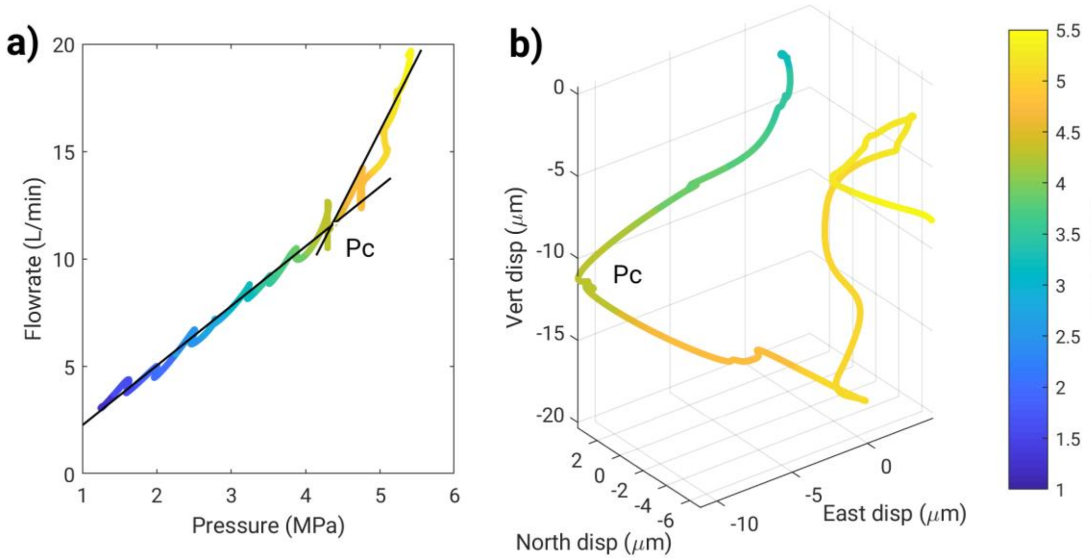

Figure S2: *Example of measure of the critical pressure Pc (Test 3, Rustrel). a) flowrate-pressure graph. Pc is the pressure where the flowrate strongly increases, i.e. for which the flowrate-pressure is no longer linear. b) 3D View of the deformation at the injection. Pc corresponds to the pressure for which the deformation switch from an elastic behavior to a plastic one, which is indicated by a sharp changes in the displacement orientation.*

Figure S3: Maximum seismic moment and monitoring parameters

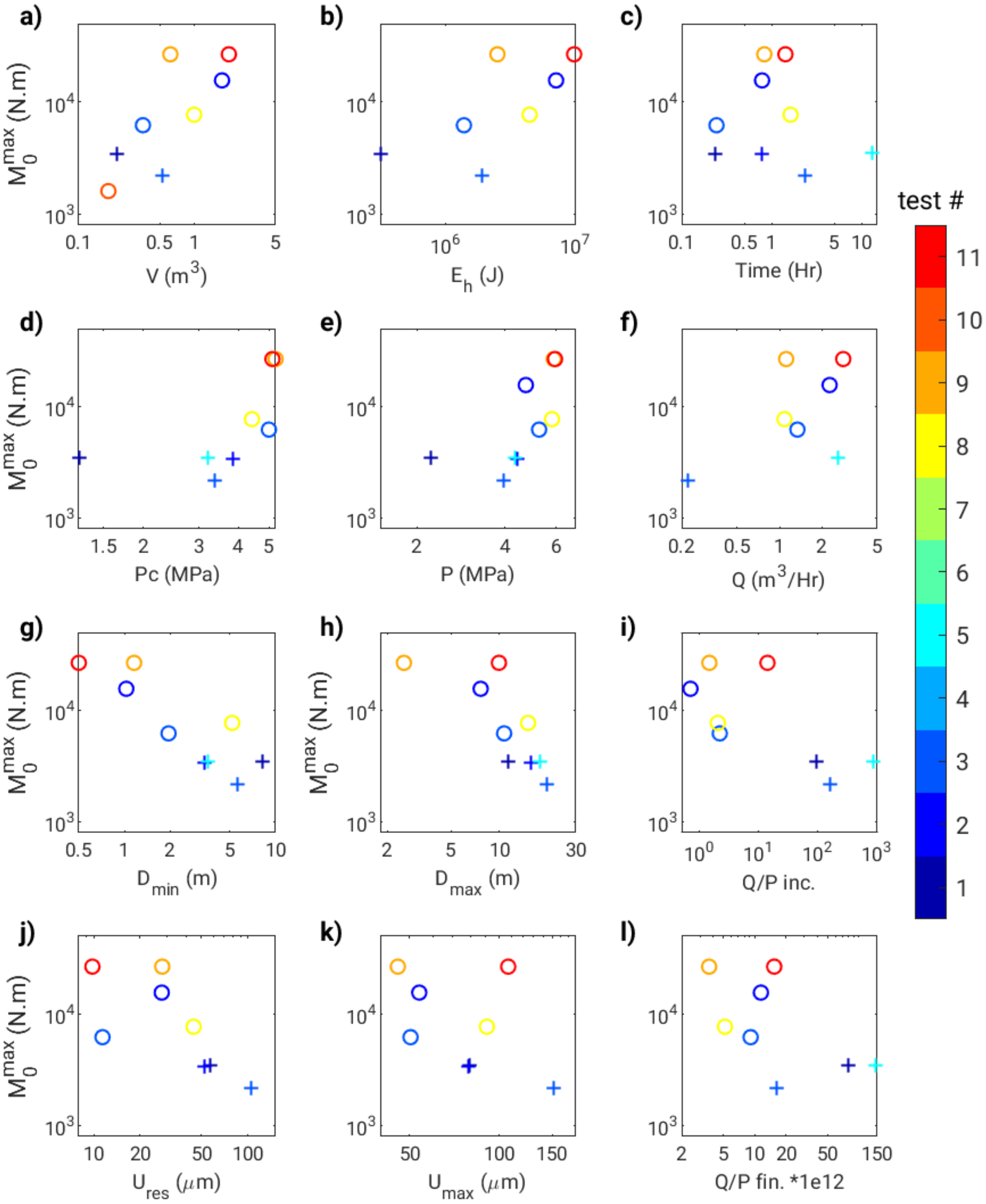

Figure S3: *Maximum seismic moment versus a) Injected volume V; b) Hydraulic energy Eh; c) Injection duration time; d) Critical Pressure Pc; e) Maximum injection pressure P; f) Maximum flowrate Q; g) Minimal hypocentral distance from the injection D_{min}; h) Maximal hypocentral distance from the injection D_{max}; i) Ratio of the final over the initial Flowrate-Pressure ratio Q/P_{inc} (i.e. proxy for increase of permeability); j) Residual displacement at the injection U_{res}; k) Maximum displacement at the injection U_{max}; l) Final Flowrate over Pressure ratio, i.e. proxy for final permeability Q/P_{fin}. Colorscale refers to the test number in either Rustrel ("o" symbols) or Tournemire ("+" symbols) experiments.*

Figure S4: Number of earthquakes and monitoring parameters

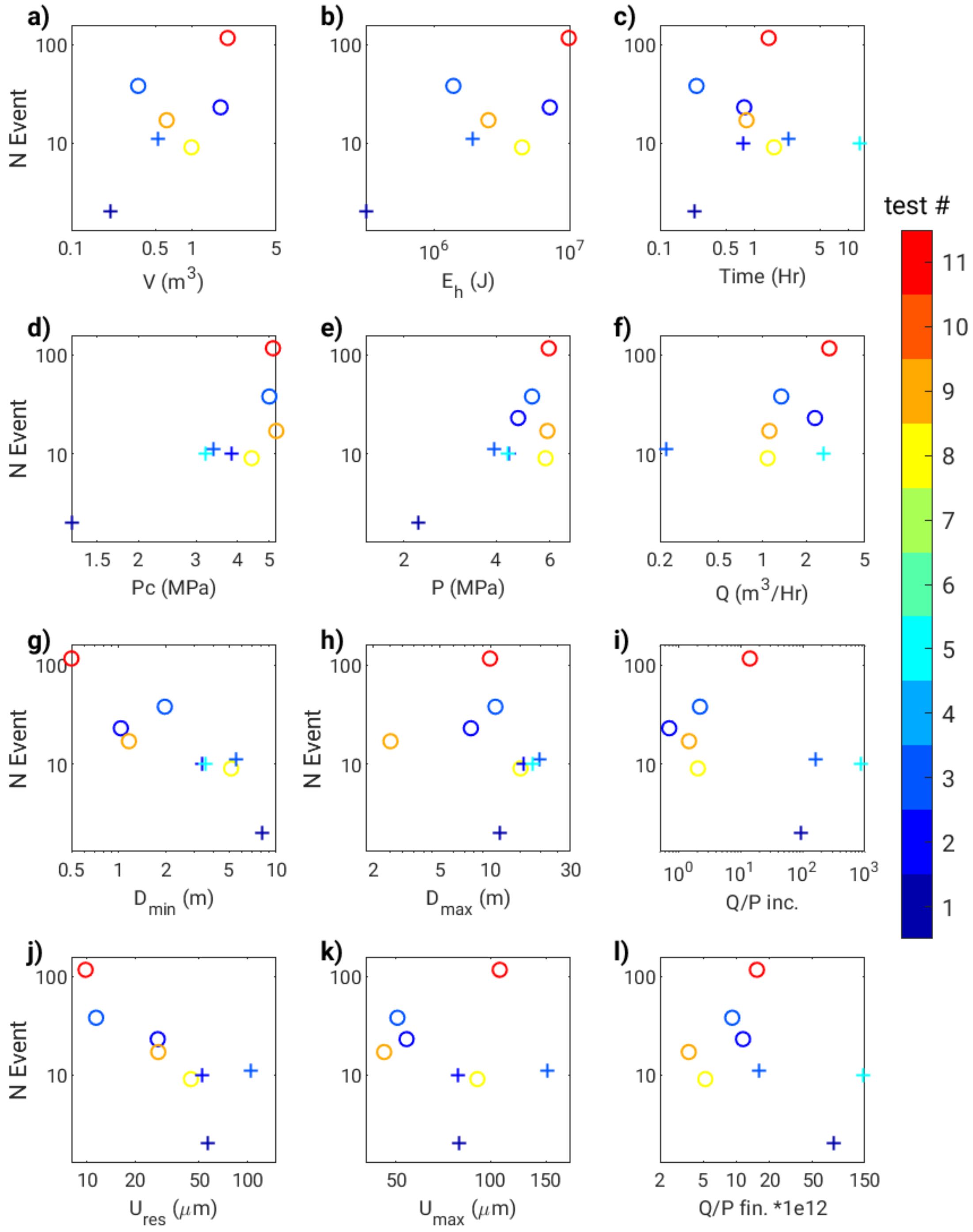

Figure S4: *Number of seismic events versus a) Injected volume V; b) Hydraulic energy Eh; c) Injection duration time; d) Critical Pressure Pc; e) Maximum injection pressure P; f) Maximum flowrate Q; g) Minimal hypocentral distance from the injection D_{min}; h) Maximal hypocentral distance from the injection D_{max}; i) Ratio of the final over the initial Flowrate-Pressure ratio $Q/P_{\ inc}$ (i.e. proxy for increase of permeability); j) Residual displacement at the injection U_{res}; k) Maximum displacement at the injection U_{max}; l) Final Flowrate over Pressure ratio, i.e. proxy for final permeability $Q/P_{\ fin}$. Colorscale refers to the test number in either Rustrel ("o" symbols) or Tournemire ("+" symbols) experiments.*

Figure S5: Trade-off among monitoring parameters

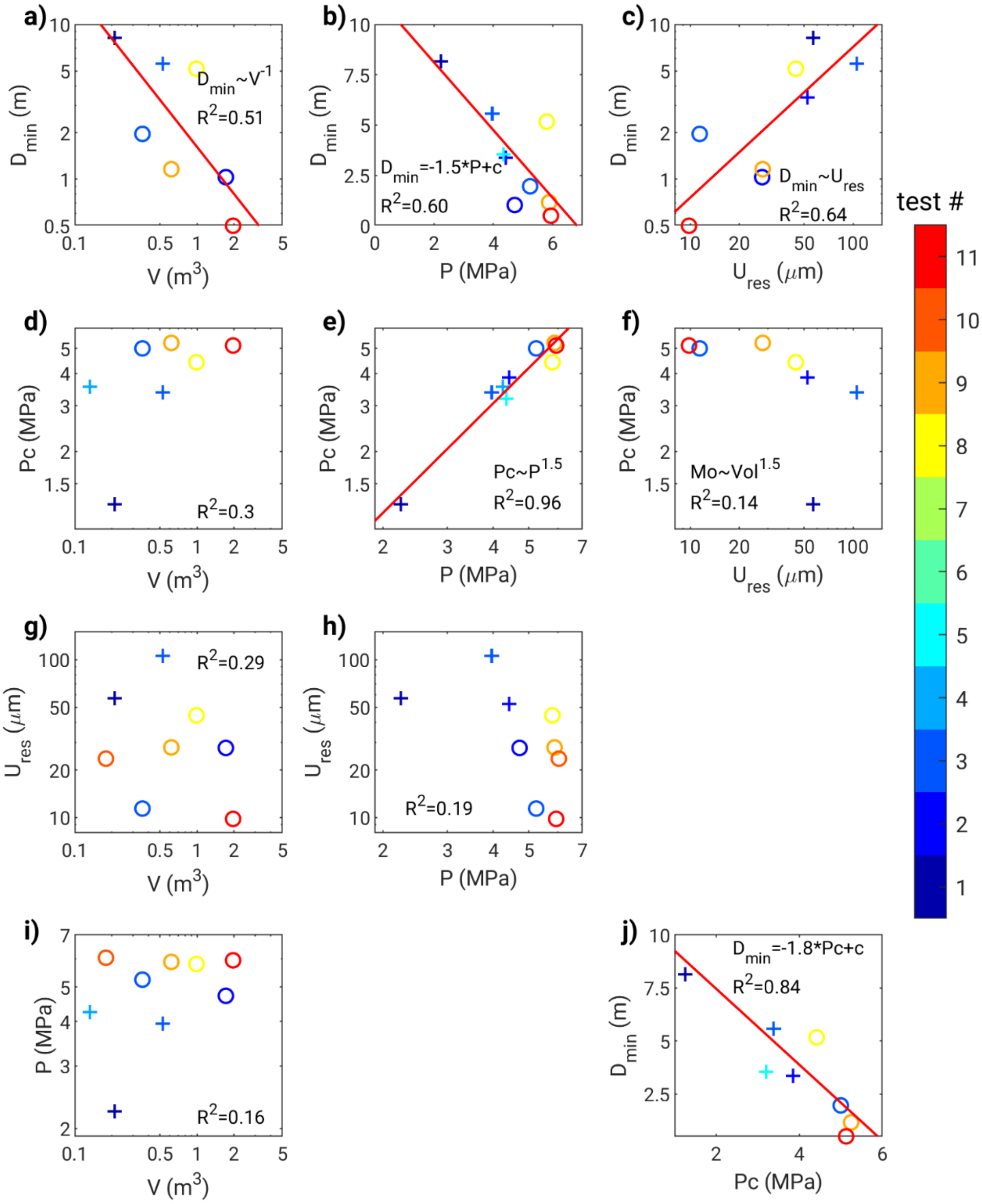

Figure S5: *Relationships among the parameters involved in the seismic energy release, namely maximum pressure P, injected volume V, residual displacement at the injection Ures, critical pressure Pc and minimal hypocentral distance to the injection Dmin. a) Dmin Vs V; b) Dmin Vs P; c) Dmin Vs Ures; d) Pc Vs V; e) Pc Vs P; f) Pc Vs Ures; g) Ures Vs V; g) Ures Vs P; i) P Vs V; j) Dmin Vs Pc. Colorscale stands for the test number, in Tournemire ("+" symbols) and Rustrel ("o" symbols) experiments . Note that b) and j) have linear axes, while the other panels show logarithmic scales. The red lines indicate the best fitting lines, when the R^2 coefficient is greater than 0.5. In this case, both R^2 and inferred relationships are given, while R^2 only is indicated in other panels.*

Table S6: monitoring data

	Test	P (MPa)	V (m^3)	Q (L/mn)	Nev	M_w^{max}	M_0^{tot} (N.m)	Pc (MPa)	Umax (µm)	Ures (µm)	Q/Pfin	Q/Pin	Eh (MJ)	Dmin (m)
Tournemire	1	2.235	0.211	NaN	2	-3.84	3987	1.25	80	57	4.7	0.05	0.31	8.15
	2	4.418	NaN	NaN	10	-3.85	11335	3.85	79	52	NaN	NaN	NaN	3.36
	3	3.948	0.524	3.7	11	-3.97	13962	3.38	151	105	0.97	0.006	1.91	5.58
	4	4.25	0.132	3.8	0	NaN	NaN	3.55	544	527	0.72	0.006	0.51	NaN
	5	4.35	21.26	43.6	10	-3.84	12112	3.2	NaN	NaN	8.7	0.01	8.62	3,55
Rustrel	1	1.97	2.3	67.82	0	NaN	NaN	NaN	28.5	16.5	3.5	4.3	NaN	NaN
	2	4,72	1.724	38.04	23	-3.12	60100	NaN	54	27.6	0.69	0.98	7.05	1.03
	3	5.24	0.358	22.39	38	-3.57	67350	5	50.5	11.4	0.55	0.25	1.38	1.96
	4	NaN	NaN	NaN	0	NaN	NaN	NaN	NaN	NaN	NaN	NaN	NaN	NaN
	5	1.58	1.17	67.27	0	NaN	NaN	NaN	25.5	25.1	4.6	4.7	NaN	NaN
	6	2.42	1.19	69.3	0	NaN	NaN	NaN	24.4	19.2	2.9	3.1	NaN	NaN
	7	1.86	0.77	69.81	0	NaN	NaN	NaN	14.6	6.7	3.8	4	NaN	NaN
	8	5.8	0.99	18.1	9	-3.5	17500	4.42	90.8	44.4	0.312	0.153	4.41	5.17
	9	5.88	0.617	18.6	17	-3.14	73100	5.25	45.8	27.8	0.22	0.15	2.49	1.16
	10	6.04	0.18	7.5	1	-3.96	1600	NaN	87.6	23.6	0.125	0.136	0.66	NaN
	11	5.94	1.975	47.6	117	-3.14	281000	5.13	107	9.8	0.925	0.066	9.7	0.5

Table S6: *Main monitoring parameters used in this study for the 5 tests of Tournemire (Shale) and the 11 tests in Rustrel: maximum pressure (P), injected volume (V), maximum flowrate (Q), Number of seismic events (Nev), maximum magnitude (M_w^{max}), cumulated seismic moment (M_0^{tot}), critical pressure (Pc) maximum displacement at the injection (Umax), residual displacement after the injection (Ures), final flowrate-to-pressure ration (Q/Pfin), initial flowrate-to-pressure ration (Q/Pin.), hydraulic energy (Eh), minimal hypocentral distance to the injection (Dmin).*
